# Regulatory Design Governing Progression of Population Growth Phases in Bacteria

**DOI:** 10.1371/journal.pone.0030654

**Published:** 2012-02-21

**Authors:** Agustino Martínez-Antonio, Jason G. Lomnitz, Santiago Sandoval, Maximino Aldana, Michael A. Savageau

**Affiliations:** 1 Departamento de Ingeniería Genética, Cinvestav, Irapuato, Guanajuato, México; 2 Biomedical Engineering Department, University of California Davis, Davis, California, United States of America; 3 Instituto de Física, Universidad Nacional Autónoma de México, Cuernavaca, Morelos, México; John Innes Centre, United Kingdom

## Abstract

It has long been noted that batch cultures inoculated with resting bacteria exhibit a progression of growth phases traditionally labeled lag, exponential, pre-stationary and stationary. However, a detailed molecular description of the mechanisms controlling the transitions between these phases is lacking. A core circuit, formed by a subset of regulatory interactions involving five global transcription factors (FIS, HNS, IHF, RpoS and GadX), has been identified by correlating information from the well- established transcriptional regulatory network of *Escherichia coli* and genome-wide expression data from cultures in these different growth phases. We propose a functional role for this circuit in controlling progression through these phases. Two alternative hypotheses for controlling the transition between the growth phases are first, a continuous graded adjustment to changing environmental conditions, and second, a discontinuous hysteretic switch at critical thresholds between growth phases. We formulate a simple mathematical model of the core circuit, consisting of differential equations based on the power-law formalism, and show by mathematical and computer-assisted analysis that there are critical conditions among the parameters of the model that can lead to hysteretic switch behavior, which – if validated experimentally – would suggest that the transitions between different growth phases might be analogous to cellular differentiation. Based on these provocative results, we propose experiments to test the alternative hypotheses.

## Introduction

Biological systems have multiple mechanisms to correctly self-reproduce in a manner compatible with the environment in which they exist. In the cell cycle of eukaryotes these are the checkpoints that are identified with “periodic genes” [1], [2]. In the cell cycle of prokaryotes, however, the evidence indicates a continuous process without such checkpoints [3]. It has been known for some time that bacterial batch cultures tend to follow a well-defined progression of growth phases: lag, exponential, early stationary and stationary [4]. The first reports characterizing population growth of bacteria in cultures were published about 90 years ago [5]. Since these first studies, different growth phases have been identified and modeled [6]. Subsequent reports described physiological states associated with these growth phases [7], [8]. Different growth phases correspond to a more or less well-defined metabolism and physiological status: Adaptation of cellular machinery to new environmental conditions in lag phase; maximal growth rates in exponential phase; slowing of metabolic rate by nutrient deprivation or stressing conditions in early stationary phase; and arrest of metabolism and implementation of a resistant physiology in stationary phase. The question we address here is the following: Are there identifiable regulatory mechanisms at the single-cell level that account for the coordination of this population-level behavior?

Here we study the global regulatory principles that govern the natural progression of population growth as revealed in a batch culture of *E. coli*, the model organism par excellence. This generic progression is manifested when stationary cultures are introduced into a nutrient-rich milieu and the population is allowed to grow until cells arrest their growth due to nutrient deprivation or accumulation of toxic products. There are of course a number of more specific regulatory mechanisms at the metabolic and genetic level that can be invoked in specific circumstances and in response to transient perturbations. However, here we are concerned with the global regulation of the generic progression of growth phases as described above.

In this study we take advantage of the wealth of individual experimentally-validated regulatory interactions in *E. coli* [9] to identify an integrated system of interactions, or transcriptional regulatory circuit (TRC), whose expression patterns might be involved in the control of the population-level behavior. Mathematical and computer-assisted analysis is employed to uncover further implications of this circuit. This analysis is intended as a guide to aid our comprehension of the mechanisms controlling the phases of population growth for single-celled organisms and to suggest critical experiments for distinguishing among alternative hypotheses.

## Results and Discussion

### A transcriptional regulatory circuit controlling the progression of growth phases

**Figure 1:**
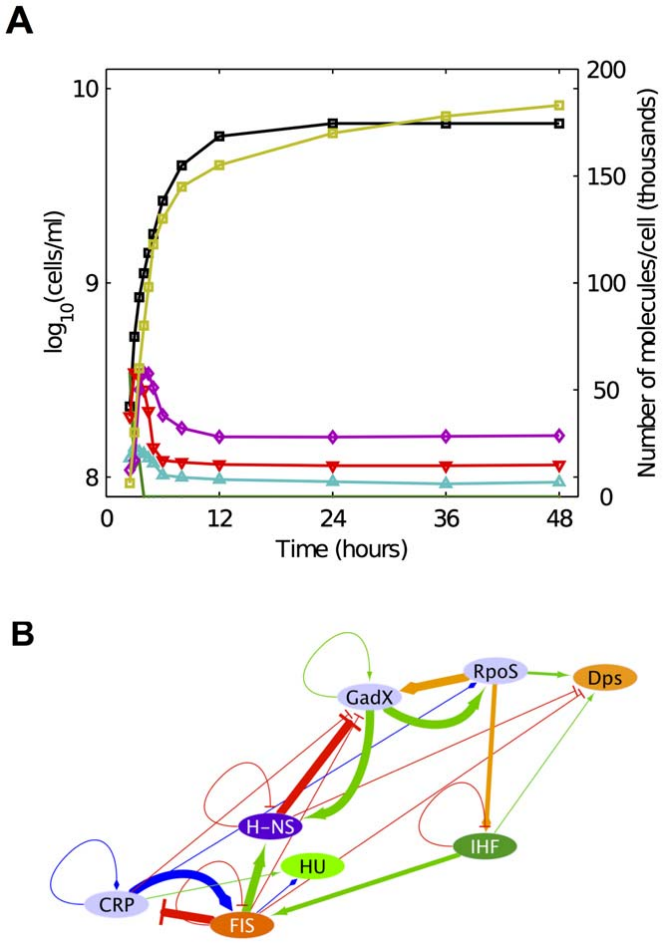
Progression of growth phases in an *Escherichia coli* culture. A) Expression levels of different NAPs (see Table 1) in a culture growing exponentially in a rich medium and following a down-shift to a nutritionally-depleted medium at time zero; the black line shows the growth curve. Note that this data does not include the lag phase or the transition to the exponential growth phase. The number of molecules per cell for the transcription factors are as follows: Dps (yellow), HU (magenta), HNS (red), IHF (cyan), and FIS (green). This figure was drawn with data from Figures 1 and 3 of Ali Azam et al. [10]. B) The transcriptional regulatory circuit (TRC) involving TFs regulating *E. coli* growth phases: Green edges represent activation, red repression, yellow transcription by sigma32, and blue dual regulation (both activation and repression). Thicker lines are used to emphasize the interactions studied in this work.

Ten years ago several nucleoid-associated proteins (NAPs) were reported to reach maximal expression levels at different growth phases of *E. coli* [10] (Figure 1A). As a result of recent genome-wide binding studies it is now postulated that NAPs, in addition to restructuring the bacterial nucleoid in *E. coli*, influence global transcriptional programs [11]–[13]. This is possible because NAPs are highly abundant small proteins in the cell that bind DNA without a clear DNA-sequence consensus. Depending on the types of NAPs, the bacterial nucleoid can be potentially restructured in different ways, enabling distinct global transcriptional programs [12]. Hence, it is proposed that NAPs exert an analog-like control on gene expression that is complementary to the digital-like control exerted by most sequence-specific DNA-binding transcription factors (TFs) [11], [14]. The TRC in Figure 1B suggests that, in addition to the three main NAPs defined as global regulators [15] (Factor for Inversion Stimulation—FIS; Histone-like Nucleoid Structuring protein—HNS; and Integration Host Factor—IHF), two other components play an important role in controlling the growth-phase transitions. One of those components is a general stress-resistant sigma factor (RpoS) [16], and the other is an acid-stress regulatory protein (GadX) [17] (see Table 1).

**Table 1:** Brief description of TFs in the circuit.

TF	Description
**FIS**	(Factor for Inversion Stimulation) is a 22 kDa homo-dimeric protein. FIS binding results in DNA bending between 50° and 90°. FIS is the most abundant NAP in early exponentially growing cells (1 Fis/450 bp) [50] and references therein.
**H-NS**	(Histone-like Nucleoid Structuring protein) is a 15.4 kDa protein conserved among Gram-negative bacteria. H-NS–DNA complexes show that binding of H-NS results in bridges between adjacent DNA duplexes providing a structural basis for their repressive role. *In vivo* over-expression of H-NS results in highly compacted nucleoids. In exponentially growing cells there is approximately 1 H-NS dimer per 1400 bp of DNA [50] and references therein.
**GadX**	(regulator of Glutamic Acid Decarboxylase) this system reaction contributes to pH homeostasis by consuming intracellular H^+^ and producing gamma-aminobutyric acid [51].
**RpoS**	(sigma factor also know as sigma S or sigma32) is a RNA polymerase subunit for stress and stationary phase transcription. It was found that sigma RpoS increases to 30% of the level of sigma 70 during transition to the stationary phase [52], [53].
**IHF**	(Integration Host Factor) is composed of α and β subunits of 11 and 9.5 kDa respectively and both share 25% identity. IHF bends the DNA and reduces chromosome length by 30%. Expression of IHF is maximal during early stationary growth (1 IHF/335 bp) [50] and references therein.

It is worth observing that the specific ordering of the regulatory interactions between the NAPs of this circuit reflects the order in time at which they are maximally expressed during the progression of growth phases (Figure 1A). The proposed circuit can help us understand how a molecular mechanism at the single-cell level might affect the emergence of phenotypic traits at the population level, particularly with regard to the transitions of the population through the different growth phases in a culture.

### Operation of the transcriptional regulatory circuit

It is well known that FIS is maximally expressed in the lag phase to activate important promoters such as those driving the expression of ribosomal genes [18], and its localization is enriched in chromosomal zones of highly expressed genes, as revealed by recent studies of genome-wide localization [19], [20]. In a similar way, HU (Histone-Like) and H-NS have been found maximally expressed during the exponential phase of growth. HU is found to regulate the transcription of no more than 10 genes and it is possible that its role could be more structural than regulatory. On the other hand, H-NS represses a large number of genes during the exponential phase and is implicated in the silencing of horizontally acquired genes and pseudogenes, also revealed by genome-wide localization analysis [21]. The activity of the TRC formed around *gadX* (Figure 1B) appears to control the critical transition from a growing to a growth-arrested population. In a growing culture the negative influence of H-NS on transcription of *gadX* should maintain GadX at its lowest levels and prevent the activation of *rpoS*, the gene encoding the sigma factor for stationary phase.

**Figure 2:**
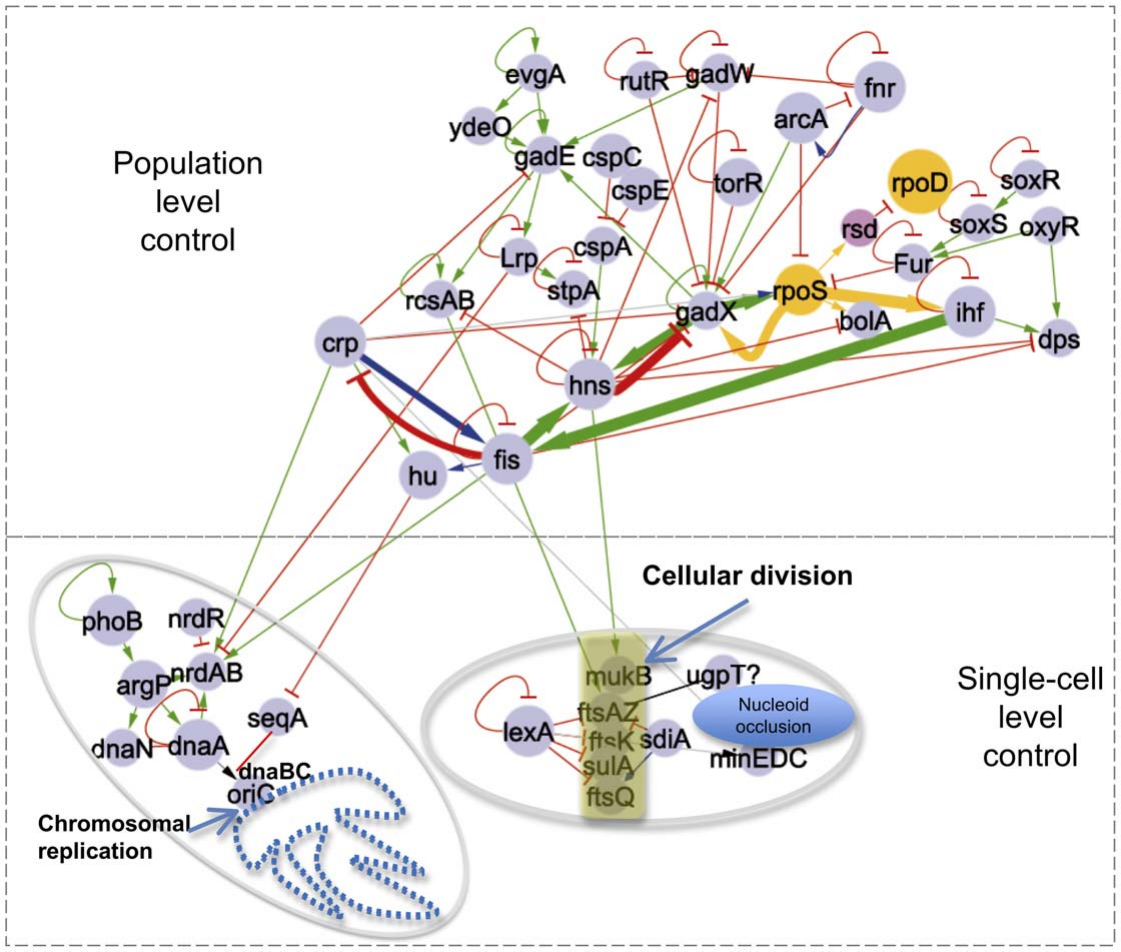
The regulatory network coordinating growth at the population level with chromosomal replication and cell partitioning in *E. coli*. The players shown are those for which there is some transcriptional information available. The lower parts represent the molecular events at the cellular level (chromosomal replication and cell division), whereas the upper part represents the members of the transcriptional regulatory circuit controlling the progression of growth phases at the population level. The color code for the edges is the same as in Figure 1B.

Additional regulators also control expression of *gadX*, mostly those regulating respiratory processes (Figure 2). Thus, once their threshold is exceeded, (principally by media acidification due to accumulation of waste products, such as acetate, generated by active metabolism), GadX levels increase and activate *rpoS*. Since *gadX* has a promoter for RpoS, their mutual activation constitutes a robust positive circuit raising the levels of RpoS. Once a substantial part of the transcriptional control is taken over by the sigma factor RpoS, three things happen: (i) RpoS redirects the transcriptional activity of RNA-polymerase and, at the same time, blocks the activity of the housekeeping sigma factor RpoD (through the transcription of the anti-sigma factor RSD [22]), (ii) it promotes the change of cell morphology to a smaller and more resistant form (through the stationary-phase morphogene BolA [23]), and (iii) it transcribes the subunits of the global NAP IHF [24], which in turn activates Dps (DNA protection during starvation) [25]. (This last NAP is highly abundant in cells that have long been in stationary phase; its main function might be protection of DNA since to date it has not been associated with gene regulation). Additionally, FIS and H-NS, both active in the growing phase, repress jointly *gadX* and *dps*, two members of the growth-arrested regulatory machinery that should be off whenever the cell is actively growing.

The signal for resumption of growth in an arrested cell is via the activation of *fis* by IHF and CRP (cAMP Receptor or Catabolic Repressor Protein). Activation by CRP happens when a rich carbon source is present; although the condition that controls activation by IHF is less clear; IHF is involved in the process of DNA replication by bending the region of replication initiation. Overall, this transitional step from an arrested to a growing culture is the least understood part of the circuit.

### Coupling of bacterial growth with chromosomal replication and partitioning of the cell

Until now we have described a circuit formed by TFs whose activity plays an important role in controlling the transitions between a growing and an arrested culture. However, we know that a growing culture results from the division of individual cells. Bacterial cell division depends on a series of critical control mechanisms, including those for chromosomal replication and partitioning of the cell to ensure that each daughter cell contains an uncorrupted nucleoid. Current knowledge about the molecular mechanisms controlling these processes has been thoroughly reviewed [26]–[28]. Here we ask if the transcriptional regulatory circuit controlling cell growth is also regulating the elements that control chromosomal replication and partitioning of the cell. As shown in Figure 2, there are in fact transcriptional interactions between members of the circuit controlling cellular growth and the machinery for chromosomal replication and partitioning of the cell. Moreover, we find that the only TFs of the circuit that are connected to these processes are those functioning in an actively growing culture, i.e. FIS, HU and H-NS (Figure 2). FIS and CRP activate the *nrdAB* genes [29], whose products make available the deoxyribonucleotides for DNA replication. HU represses *seqA* [30], whose product competes with DnaA for the low-affinity sequences in the DNA, and when bound prevents DnaA from initiating chromosomal replication. On the other hand, FIS and H-NS activate the main players for the cell-partitioning process. FIS promotes the production of FtsAZ proteins that form the filament at the middle of the cell during partitioning, and H-NS activates the synthesis of MukB, an auxiliary factor also involved in this partitioning mechanism [27]. Therefore, there is indeed a clear regulatory interaction between our proposed circuit controlling cell growth and the elements that control chromosomal replication and partitioning of the cell. It is important to mention that all the interactions from the transcriptional regulatory circuit that influence genes directing chromosomal replication and cell-division are positive, as should be expected, for chromosomal replication and cell partition are processes that need to be activated in a growing population.

### Mathematical model of the core transcriptional regulatory circuit governing the progression of growth phases

The transcriptional regulatory circuit described in the previous sections has many intuitive properties consistent with it being the core mechanism regulating the progression of growth phases. However, a more rigorous analysis would provide additional support for the proposed role of this circuit as well as a deeper understanding of key quantitative design issues. In this section, we describe a mathematical model that highlights key interactions in the circuit, we identify critical alternatives potentially associated with the transitions between exponential growth and stationary phases, and we make experimentally testable predictions regarding the alternative designs governing the transition between the growth phases.

**Figure 3:**
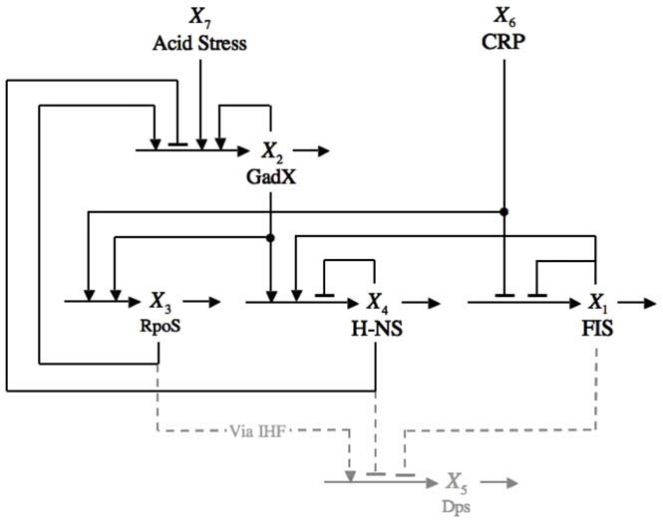
Schematic diagram of the transcriptional regulatory circuit showing the five key regulators and the interactions among them. Horizontal arrows represent mass flow by chemical reaction or diffusion. Vertical arrows pointing to horizontal arrows represent the influence of a regulator on the rate of a target process; arrows with a barbed head represent activation of the process, whereas arrows with a flat head represent repression. The arrows leading to and from Dps, a reporter of the stationary phase, are represented in gray. CRP-cAMP levels represent carbon source availability and acid/oxidative stress is represented by the activation of GadX. Activation of Dps by RpoS is done indirectly via IHF, which has been intentionally left out of the model (see text).

Our model [represented in Figure 3 and Eqs (1) in **Materials and Methods**] is formulated around four critical elements FIS, GadX, RpoS and H-NS forming a network of embedded positive and negative feedback loops. These feedback loops, and their mutual interrelations, play a key role in dictating the overall behavior of the circuit, as will be described below.

The embedded circuits respond to different stresses by means of diverse interactions. The FIS protein, which is tightly correlated with the exponential growth phase [10], activates the production of H-NS. Conversely, the effective concentration of RpoS peaks during stationary phase by means of an increased stability of the protein and various transcriptional and translational effects [31], [32]. This model varies slightly from the proposed circuit in three respects. First, we are representing the complex regulatory control influencing RpoS expression, in response to nutrient availability, as a direct influence of CRP on RpoS, which is represented by the power-law function 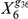. As a result, we model FIS and RpoS as proteins responding in a reciprocal fashion to carbon-source availability or milieu conditions. Second, we are adding Dps, which protects the cell from DNA damage through DNA-Dps co-crystals [33], [34], to provide a reporter that is expressed during stationary phase in response to Fis, HNS and IHF. Third, we are only including IHF implicitly at this point because the particular role of IHF in this circuit is not yet fully understood and because it lies on the periphery of what we regard as the pivotal elements of the circuit. It is known that Dps is maximally expressed in stationary phase, which suggests a positive overall influence of RpoS on Dps. Thus, we have represented this overall influence of RpoS on the rate of production of Dps by the power-law function 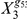 in Eqs. (1) and Eqs. (2). The inclusion of Dps allows us to qualitatively compare our results with experimental profiles of NAP concentration in the different growth phases [10]. The last element of the model is the activation of GadX production as a result of either acid stress or oxidative stress, both of which can drive the cell into stationary phase [35], [36].

### Design space and steady-state analysis of the model

The model described in the previous section can be recast exactly into the standard Generalized Mass Action (GMA-system) representation within the power-law formalism [37]. The qualitatively distinct phenotypes of such a model can be identified with distinct combinations of ‘dominant processes’ for the synthesis and degradation of each species in the GMA model [38], [39]. Each such combination corresponds to a potentially valid *synergistic* or *saturable* (S-system) representation within the power-law formalism. These nonlinear differential equations have an analytical solution for their steady states, which is not the case for most nonlinear differential equations. Moreover, by taking logarithms, the steady-state equations can be transformed into a set of linear algebraic equations with a familiar solution [37], [40], [41]. The conditions implied by a selection of dominant processes correspond to a set of linear inequalities in log space that, together with the corresponding steady-state solution, specify the boundaries within which the S-System is valid [38], [39], [42], [43]. In general, the number of combinations of dominant terms provides a bound on the number of qualitatively distinct phenotypes [38]. The number of equations and terms in our recast model yields a maximum of 256 qualitatively distinct phenotypes, which are numbered arbitrarily, but only 21 of these are valid. By careful inspection of the valid cases, we are able to distinguish qualitatively distinct phenotypes that are associated with the proposed consortium of global transcription factors. In the following paragraph, a few cases are highlighted to show their agreement with well-known phenotypes, such as quiescent and growing cells.

The two most extreme cases, associated with either exponential growth or stationary phase, are Case 193 and Case 64. In Case 193, both GadX and RpoS are expressed at their *maximal level*. This response, as described previously, is induced by either carbon source starvation or through the acid/oxidative stress response, and thus is consistent with a stationary-phase phenotype. In contrast, Case 64 represents the situation in which GadX and RpoS are expressed at their *basal level*. This response, as described previously, is consistent with an exponential-growth phenotype. Other cases involve mixed responses to the two types of stress or partial induction of a stress response.

The strength of this approach lies in its ability to capture qualitatively distinct global behavior with rigorous and analytically tractable methods. Thus, we can study the system without necessarily specifying particular parameter values, which allows us to explore the full phenotypic repertoire of the proposed model. With this approach we are able to identify global tendencies without relying on precise parameter values.

### Local stability analysis of GadX expression in its regulatable region

The behavior of the circuit when all transcription-factor targets are in their regulatable region (neither completely saturated nor completely unsaturated) is critically dependent on the levels of the GadX protein: as the concentration of GadX increases, both negative and positive feedback effects occur. The outcome of these interactions is not obvious, and a number of qualitatively distinct behaviors may appear as different interactions dominate.

To address these issues we have analyzed the local S-system [Eqs. (4)] representing the most general situation in which all transcription-factor targets are in their regulatable region and the system is operating around the intermediate steady state. Because of the tractability of these equations [37] we are able to analytically determine a mathematical condition [Eq. (5) in **Materials and Methods**], expressed in terms of the kinetic orders, that indicates instability of the intermediate steady-state solution. Interestingly, this condition reflects a relationship among the feedback loops affecting GadX expression. If the relationship reveals a net dominance of the positive feedback effects over the negative feedback effects, then the steady state becomes unstable in a manner characteristic of hysteretic switches. This hysteretic behavior implies a differentiation-like response: when a change in signal (*e.g.*, acid stress) reaches a threshold value, the cell commits to a new physiological state, and once committed the process of reverting to the original state requires a greater change of signal in the opposite direction [37], [42]. The advantages of the “buffer zone” created by hysteretic switches are well known. It can protect the cell from inappropriately rearranging its physiology in response to minor fluctuations in the environment, which would lead to unproductive rearrangements of the cell's protein profile that can be both energetically taxing and time consuming. Furthermore, there are critical moments for cell survival when the environment becomes detrimental because of nutrient depletion and/or other stresses, and the cell cannot afford to vacillate between growth and stationary phases.

Alternatively, the condition for hysteresis might not be satisfied and the stable steady state would then be indicative of a system with a continuous incremental adjustment to changing environmental conditions. A relevant advantage of such a mechanism, when compared to a hysteretic one, is a faster response to small changes in environmental conditions. These two types of responses may be considered the manifestation of alternative strategies for dealing with the stresses associated with population growth.

### Comparison of the alternative strategies

Analysis of the model has revealed two distinct strategies for controlling the transition between the growth phases. The two alternatives, as mentioned in the above section, are a continuous graded response and a discontinuous hysteretic switch. The two strategies show complementary qualities: the continuous response favors a gradual adjustment to changes in environmental conditions, whereas, the discontinuous response favors a more invariant cellular state until critical thresholds are reached.

In this section, we describe a well-controlled comparison [37] of the alternative strategies in models that are identical, except that the condition in Eq. (5) is satisfied for one alternative and not the other. For example, we ensured that the capacity for regulation of each transcript by each of the regulators was the same for both alternatives; we also used the same values for the corresponding parameters, with the exception of one kinetic order (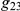) that was changed to satisfy/violate the condition for hysteresis. Moreover, the two alternatives were chosen such that the switching effort [44] – defined as the magnitude of the stimulus required for the transition between a growth-state to a quiescent-state, or vice versa – was the same.

At this point we note that the condition in Eq. (5) tends to favor the hysteretic strategy. If realistic parameter values are used for the kinetic orders, assuming first and second order kinetics, the inequality tends to be satisfied. In order to violate the inequality the magnitude of the parameter 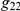, representing the positive auto-regulation of *gadX*, must be made small for a graded response. In our comparison, first-order kinetics were assumed for all interactions except for two that were made different in order to match the switching effort of the two alternatives. A kinetic order of two was assumed for the repression of *gadX* by HN-S in order to favor the net negative feedback, and a fractional kinetic order was assumed for the positive auto-regulation of *gadX* in order to dampen the net positive feedback.

Although the design associated with hysteresis appears more likely by inspection of the condition in Eq. (5), there has been no empirical evidence to support the existence of a developmental switch-like mechanism governing the progression of growth phases in *E. coli*. To address this issue, we (1) compared the alternative designs for their ability to successfully reproduce known experimental data and (2) provided analytical results that suggest critical experiments to distinguish between the alternatives.

**Figure 4:**
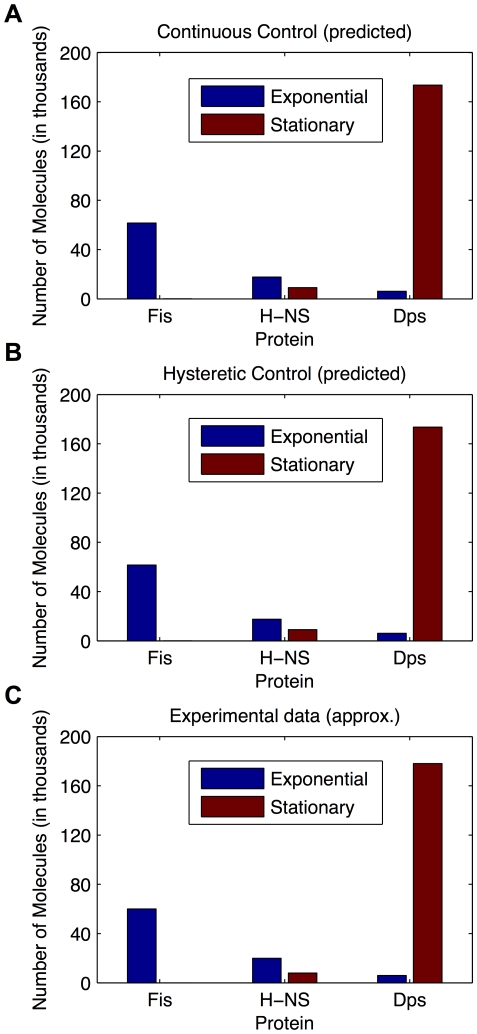
Number of molecules for three proteins, Fis, HNS and Dps. For each regulator, data is presented in a pair: the first bar represents exponential growth phase (blue) and the second bar represents stationary growth phase (red). A) Predicted results for the system with a continuous graded phenotype. B) Predicted results for the system with a discontinuous hysteretic phenotype. C) Approximate experimental values. The experimental data were drawn from Figure 3 of Ali Azam *et al.* [10].

First, we compared the alternatives under conditions analogous to those used in generating the experimental data, in particular the data reported by Ali Azam, *et al.* [10] for the transition from exponential growth to stationary phase. We calculated the number of molecules at steady state for the two systems in the regions of design space representing exponential growth and stationary phase (outside the regions of transition), and compared the calculated data with the experimental data (Figure 4). The results show that the alternative strategies are effectively identical in this respect and, consequently, either design could potentially reproduce the experimental data of Ali Azam, *et al.* [10].

**Figure 5:**
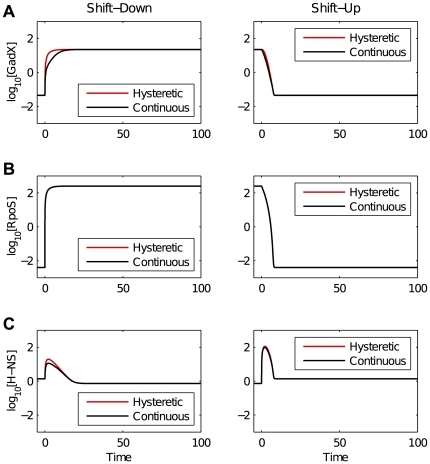
Simulated dynamics for the system exhibiting a continuous graded phenotype (black) and for the system exhibiting a discontinuous hysteretic phenotype (red). Responses are shown for an instantaneous change in the medium: rich to poor (left panels) or poor to rich (right panels). A) Response for GadX. B) Response for RpoS. C) Response for HNS. Data are qualitatively consistent with experimental data found in Ali Azam, *et al.* [10].

We also compared the dynamic response of the alternative designs to large changes in the environment, simulating the transition of cells from quiescence to exponential growth and vice versa (Figure 5). The alternatives showed the same qualitative behavior and were practically indistinguishable in the case of many of the transcription factors. Both were qualitatively consistent with the quantitative western blot analysis reported by Ali Azam, *et al.* [10]. The comparison between the two alternatives has shown that either could account for known experimental data; hence, conventional growth experiments may have difficulty revealing the differences between the alternative designs.

**Figure 6:**
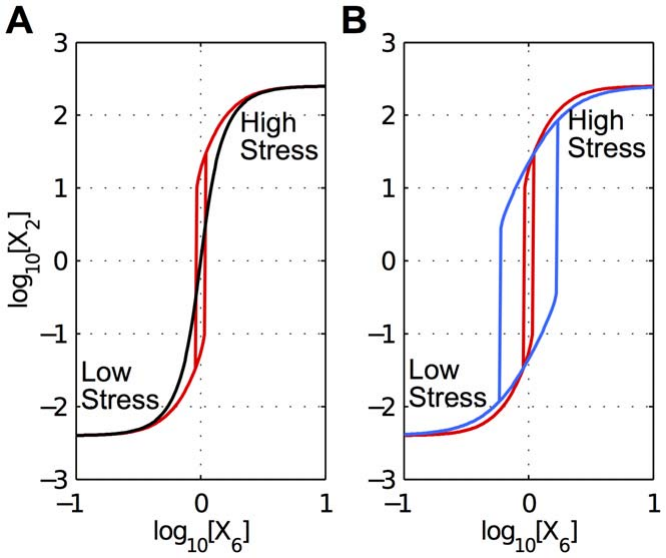
Steady-state switching characteristics for continuous and discontinuous responses. A) Switching characteristics for RpoS under the alternative hypotheses yielding a continuous graded response (black) or a discontinuous hysteretic response (red). The low-stress condition is on the left; whereas the high-stress conditions is on the right. The switching effort, which occurs between these conditions, was matched empirically for the two designs. The plot is shown for a slice of design space corresponding to the normalized value for acid/oxidative stress. B) The discontinuous hysteretic response in A) compared with another, with a wider hysteretic region (blue), for which a comparable continuous graded response is precluded.

Second, we analyzed the steady-state switching characteristics for the two designs (Figure 6). In a simulated experiment, cells initially in a quiescent state were inoculated into a set of growth chambers with a graded concentration of nutrients and allowed to achieve steady-state growth (for at least 5 generations). The concentrations of the various transcription factors were recorded for the cells in each culture. In another simulated experiment, cells initially in exponential growth were inoculated into a set of growth chambers with the same graded concentration of nutrients and allowed to achieve a new steady-state of growth (for at least 5 generations). Again, the concentrations of the various transcription factors were recorded for the cells in each culture. The simulated results show fundamental differences between the alternative designs (Figure 6A) and validate the condition for hysteresis in our model [Eq. (5)].

Furthermore, by careful inspection of the condition for hysteresis, we have found that the requirement for a continuous graded response is considerably more difficult to satisfy with realistic parameter values, which favor a discontinuous hysteretic response. Indeed, more realistic values that enhance the discontinuous hysteretic response actually preclude the possibility of a continuously graded response with the same switching effort (Figure 6B).

**Figure 7:**
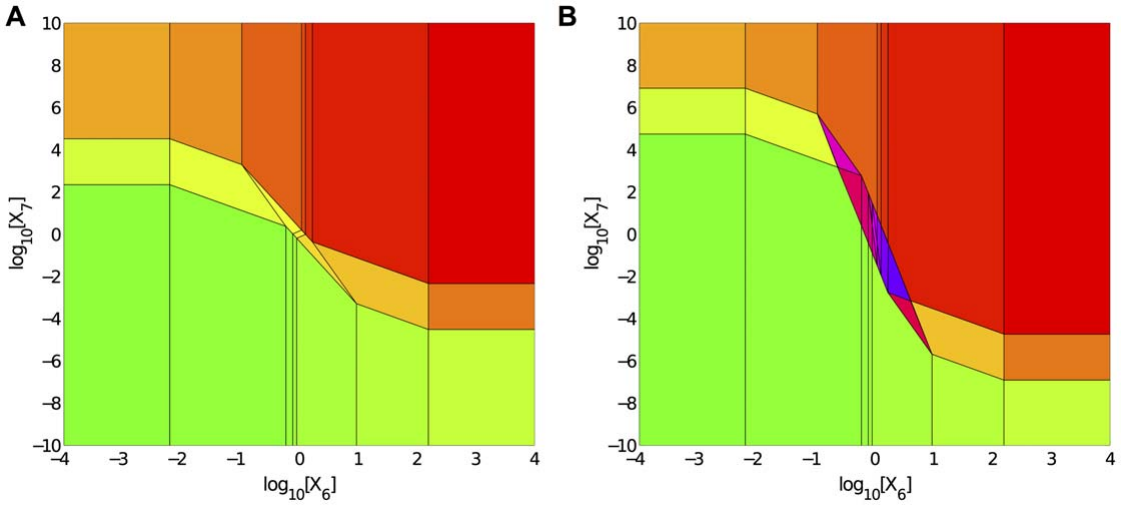
System design space displaying the qualitatively distinct phenotypes of the transcriptional regulatory circuit in **Figure 3**. Environmental stimuli are shown on the x- and y- axes. The x-axis represents carbon source depletion (low values correspond to a rich carbon source; high values correspond to a poor carbon source) and the y-axis represents acid/oxidative stress (low values correspond to low stress; high values correspond to high stress). A) System design space depicting only continuous graded phenotypes. B) System design space depicting both continuous graded and discontinuous hysteretic phenotypes. Green regions represent cases with an exponential growth-like phenotype, such as Case 256, and red regions represent cases with a stationary phase-like phenotype, such as Case 1. Purple/blue regions correspond to three overlapping cases, two stable and one unstable, indicating bistability.

To understand further the implications of the alternative designs, we analyzed their behavior in design space by examining the responses to both carbon-source depletion and acid stress (Figure 7). The design spaces of the two alternatives exhibit a large number of qualitatively distinct phenotypes that are remarkably similar (same colors), except for the regions of transition (different colors). Taken together, these results suggest that new experimental approaches involving increasing and decreasing titrations in the steady-state level of environmental stresses (such as carbon sources or organic acids), may be necessary for the critical tests required to discriminate between the alternative hypotheses.

The term “lag phase” should be clearly distinguished from the term “lag” used to describe the behavior exhibited by a culture whenever it makes a transition from one steady-state condition to another. In the classic experiments of the Copenhagen School [45] the two steady states of growth were rigorously established and the levels of cellular macromolecules were carefully measured in the transition between these states. This work included both “shift-up” (from poorer to richer media) and “shift-down” (richer to poorer media) experiments. In general, it was demonstrated that it takes about 5 generations to establish the new steady state of exponential growth. The mechanistic basis for this “lag” was elucidated by the work of Cooper and Helmstetter [46], [47], who showed that there are well defined relationships between cell mass, initiation of chromosomal replication and cell division. Namely, cell division only occurs following the accumulation of a critical ratio of cell mass to the number of chromosomal origins. Cells in poor media grow slowly and have a small size. When shifted to rich media, the rate of ribosomal synthesis increases abruptly, although the cells do not divide until the critical ratio of mass to origins is achieved. Conversely, cells in rich media grow faster and have a larger size. When shifted to poor media, the rate of ribosomal synthesis decreases abruptly, although the cells continue to divide at the former rate until the critical ratio of mass to origins is reached in the new conditions.

Intuitively, the macromolecular profile specific to the first steady state must be diluted out to fully establish the profile specific to the second steady state. Following 5 generations of growth, the original values for even the most stable molecules are reduced to ∼3% of their initial values. The response of our model to a shift-up or shift-down transition also shows a lag of about 5 generations (see Figure 5) because we have assumed stable proteins diluted by growth.

**Figure 8:**
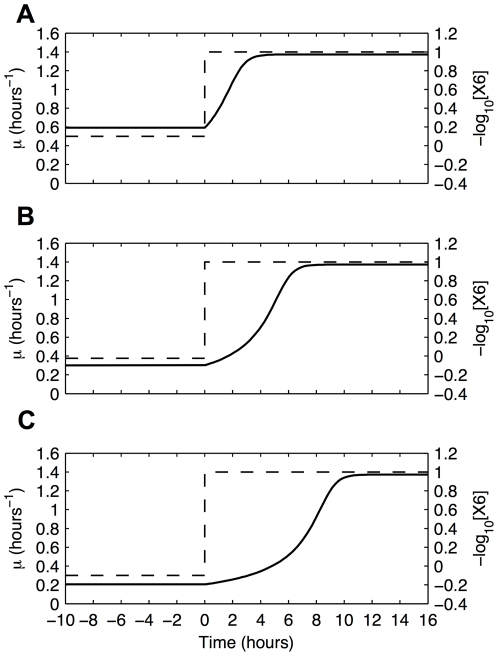
Growth rate as a function of time following a shift from nutrient-depleted medium to a rich medium at time zero. Nutrient level (dashed lines) and growth rate (continuous lines) as a function of time. (A) Minimal depletion. (B) Moderate depletion. (C) Severe depletion.

**Figure 9:**
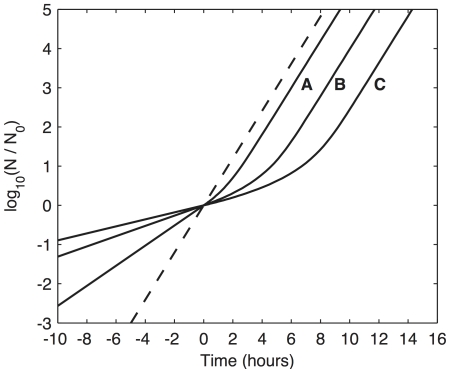
Exponential growth of cell number as a function of time. The dashed line represents a culture growing in a rich medium, washed at time zero, and re-suspended in the same rich medium. The continuous lines represent cultures experiencing a shift-up from a nutrient-depleted medium to a rich medium at time zero. The label associated with each shift-up curve corresponds to the three conditions described in the legend of Figure 8. The lags are (A) ∼1 hour, (B) ∼3 hours, and (C) ∼6 hours. (See text for discussion.)

Thus, rapidly growing cells in exponential phase are large and must first decrease in size before they can begin dividing more slowly, as is evident in Figure 1A of Ali Azam, *et al.* [10]. Conversely, slow growing cells are small and must first increase in size before they can begin dividing more rapidly. Although experimental data for the transition from lag phase to the exponential phase of growth (comparable to that of Ali Azam, *et al.* [10] for the transition from exponential growth to stationary phase) is not available, our simulations of such transitions show the expected lag before fully establishing the new growth rate. Cultures in differing states of nutrient depletion show lag periods that range from one to six hours (Figure 8). These lags are also evident in the conventional plots of exponential cell number as a function of time (Figure 9).

The reconfiguring of the expression profile for cells deep into the stationary phase or the early lag phase, when there is no growth, can be even longer than the 5 generations associated with growing cells, although these responses would undoubted involve down-stream processes that are not part of our current model.

### Conclusions

After a century of observations in which culturing bacteria leads to a reproducible progression of growth phases we report a circuit present in the transcriptional regulatory network of *E. coli* [48] that may be operating behind these visible manifestations of population growth. The main constituents of this core regulatory circuit are on the one hand elements connected to the rest of the transcriptional machinery regulating different aspects of metabolism and physiology and, on the other, the NAPs involved in conformational changes of the nucleoid that result in wide-spread effects associated with changing global transcriptional programs.

The first part of this circuit is promoting chromosomal replication and cell division in a growing population while the second part is preparing the bacterial cells for a resistant physiology in a growth-arrested culture. Even if the definitive experimental validation of this regulatory circuit remains to be done, it should be remarked that all of the pair-wise interactions among the relevant transcription factors have been experimentally established, as documented in RegulonDB.

The function of this circuit operating at the single-cell level, but with visible results at the population level, implies that bacterial populations are able to coordinate extensive transcriptional programs (representing alternative physiologies) in ways that until now have been difficult to discern. In addition to supporting this hypothesis, the qualitative results from our dynamic model identify two strategies for the operation of this circuit; the result is alternative phenotypes at the single cell level with different implications in the context of a culture. Moreover, we have found a condition among the parameters of the circuit, which we might call a ‘system design principle’, required for the manifestation of the two alternatives – either a differentiation-like process or a gradual adjustment to the environment.

The analysis of the alternative designs has shown that available experimental data does not distinguish between them. This result is consistent with the possibility of a hysteretic switch controlling the progression of growth phases, despite never having been observed. However, we have proposed critical experiments that may assist in distinguishing between the alternative hypotheses and uncovering the underlying regulatory design that governs the transition between growth phases.

Our results for this model reveal a bias toward the discontinuous hysteretic response since this response is readily obtained with realistic parameter values, whereas the continuously graded response can only be obtained with more unrealistic values. This bias might be eliminated (or reversed) if the model were to be modified significantly by the inclusion of some hypothetical negative feedback interactions.

The model presented here predicts conditions for which the transition from a growing to an arrested culture might be reversible. Since this transition has been well-studied experimentally, it should be possible to test this prediction with existing methods. However, the model predictions for transitions in the reverse direction, from an arrested to a growing culture, might require the development of new methods, since these transitions have been less well studied. Furthermore, it is well known that the associated physiology of *E. coli* in early or late stationary phases can fluctuate significantly, so it is possible that the transition from arrested to growing culture may depend critically on these fluctuations. Also, we have left out of our models some components that are known to be involved in the regulatory mechanisms controlling this transition, such as IHF, but whose precise role is still unknown.

Finally, it will be important to learn if similar circuits exist with the same basic design operating with other regulatory factors to control the population growth of organisms other than enterobacteria, since some key elements of the *E. coli* circuit, such as Gad, are not well conserved beyond this class of organisms.

## Materials and Methods

### Biological Data

The biological dataset of regulatory interactions in the circuit was obtained from RegulonDB 6.7 [9], complemented with revisions from recent literature.

### Mathematical Model

The set of kinetic equations representing the circuit in Figure 3 is the following.
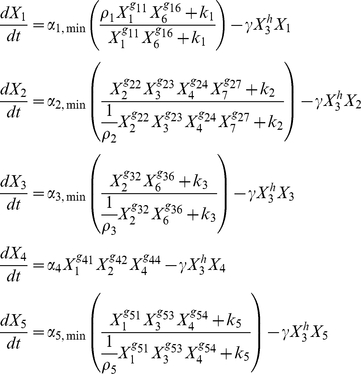
(1)The symbols are the following: 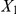 (Fis), 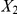 (GadX), 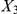 (RpoS), 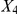 (H-NS), 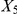 (Dps), 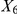 (carbon source availability), and 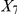 (acid or oxidative stress). The kinetic orders, 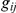, represent the influence of protein *j* on the synthesis of protein *i*, and the rate constants 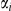 are associated with the rates of synthesis of 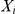. The constants 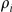 represent the capacity for regulation of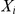. The constants, 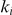, represent the half-maximal induction/repression for the expression of regulator 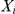. All transcription factors are assumed to experience a first-order loss with a rate constant that is grow-rate dependent, here represented by an inverse relationship (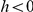) with the concentration of RpoS (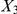). Thus, RpoS is considered a proxy for the growth rate (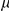); alternatively, the growth rate can be obtained from the algebraic relationship 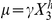 or from the set of differential equations obtained by adding the following equation to Eqs. (1):
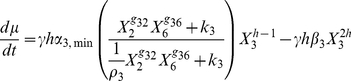
(2)and cell number as a function of time can be obtained from the solution of Eqs. (1) through (3).
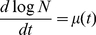
(3)


The mathematical model was developed using the piecewise power-law representation within the power-law formalism [37], [41]. The piece-wise analysis presented here is an exact representation of the circuit at three unique steady states: one extreme represents exponential growth; the other extreme represents stationary state; and an intermediate steady state within the regulatable region [Eqs. (4)], which is determined by the affinity constants, 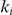. The global behavior of the circuit is a mechanism-independent extrapolation from these three steady states.

The rates of transcription and translation are collapse into a single kinetic step, which is a conventional assumption with transcription implicitly represented as a fast process. In our analysis we focus on the steady states and local stability, for which the aggregate kinetic orders of our model are the logarithmic gains of the extended model in which transcription is made explicit [40], [41]. Protein loss is assumed to be a first-order process represented by dilution in an exponentially growing culture.

The local S-system representing the circuit in Figure 3 when all transcription-factor targets are in their regulatable region is the following.
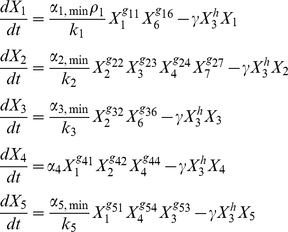
(4)The variables are the same as in Eqs. (1). The system represented by these equations is an exact representation of the circuit at the intermediate steady state, in a manner independent of the regulatory mechanisms. The local behavior in the vicinity of this intermediate steady state is guaranteed to be valid.

The inequality representing the condition for instability of the S-system in Eqs. (4) is the following.
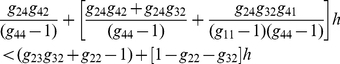
(5)The left side of the inequality represents the kinetic orders associated with the negative feedback loops whereas the right side represents the kinetic orders associated with the positive feedback loops. For unstable behavior, the positive feedback loops must dominate over the negative feedback loops. Recall that 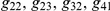 and 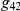 are parameters with positive values and 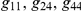 and 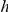 are those with negative values.

For the design space analysis, a mathematically equivalent set of steady-state equations was obtained by recasting Eqs. (1) into the generalized mass action representation in which all equations are simply sums of products of power-laws [38]. The values of the parameters were chosen, both for the simulations and for the construction of the design space, by fitting the steady states of the continuous system with experimental data. We varied the parameters over wide ranges and the qualitative nature of the global behavior did not change significantly, as long as the condition for instability/stability [Eq. (5)] was maintained (data not shown). These observations were obtained using the Design Space Toolbox in Matlab® [49].

### Fitting experimental data

The experimental data of Ali Azam, *et al.* [10] are reported as numbers of molecules per cell. The concentrations of the proteins in our simulations were converted into molecules per a normalized cell volume of 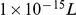. The two extreme steady states of our model, representing exponential growth and stationary phase, were fit to the experimental values obtained from cultures initially growing exponentially and then after 24 hours in stationary phase.

### Simulation of dynamic responses

The dynamic response in going from one extreme steady state to the other was simulated using the 15 s stiff ODE solver in Matlab®. The initial conditions corresponding to one of the extreme steady states was established before time zero. At time zero, we adjusted the value of the independent variable to reflect a large-scale change, and followed the dynamics until the other extreme steady state was achieved. It should be noted that the simulated growth rate changes continuously during the transient, achieving final steady-state values of 2 doublings per hour in rich media and essentially indistinguishable from zero in poor media.

### Steady-state switching characteristics

The steady states of the switching characteristics were obtained using the 15 s stiff ODE solver in Matlab®. The independent variable, either carbon source or acid stress, was set to a given value and the dynamic solution was allowed to proceed for an interval of time sufficient for the system to reach a final steady state. The final steady-state concentrations for the dependent variables were recorded and then used as the initial conditions for the next iteration following a small change of the independent variable. This iterative procedure was used to cover the entire span of values shown.

In order to demonstrate the hysteretic behavior predicted by the design space analysis, we simulated the progression of steady states in both directions: the first curve was obtained by setting the independent variable at a low value and progressively incrementing it until the upper threshold value was exceeded, whereas the second curve was obtained by setting the independent variable at a high value and progressively decrementing it until the lower threshold value was exceeded. The procedure was followed for both the design exhibiting the continuous graded response and that exhibiting the discontinuous hysteretic response.

### Design space analysis

The design space, based on the recast model previously described, was constructed using the Design Space Toolbox in Matlab® [49]. The condition for instability was found by analyzing the local stability of the intermediate steady state in which all transcription-factor targets were in their regulatable region, and applying the Routh criteria [41]. The parameters chosen were the same as those for the simulations, with the exception that the independent variables were allowed to vary over a wide range. We consider this range broad enough to cover the biologically realistic range of operation for this transcriptional circuit.
